# Antimicrobial, Anti-inflammatory, and Wound Healing Properties of *Myrtus communis* Leaf Methanolic Extract Ointment on Burn Wound Infection Induced by Methicillin-Resistant *Staphylococcus aureus* in Rats

**DOI:** 10.1155/2024/6758817

**Published:** 2024-06-11

**Authors:** Zohre Jafari, Hassan Bardania, Mehrzad Jafari Barmak, Saba Eslami, Yaser Mahmoudi-Mourderaz, Narges Roustaei, Mohammad Sharif Talebianpoor, Esmaeel Panahi Kokhdan, Seyed Sajjad Khoramrooz

**Affiliations:** ^1^ Student Research Committee Yasuj University of Medical Sciences, Yasuj, Iran; ^2^ Cellular and Molecular Research Canter Yasuj University of Medical Sciences, Yasuj, Iran; ^3^ Shiraz University of Medical Sciences, Shiraz, Iran; ^4^ Department of Epidemiology and Biostatistics School of Health and Nutrition Sciences Social Determinants of Health Research Center Yasuj University of Medical Sciences, Yasuj, Iran; ^5^ Medicinal Plants Research Center Yasuj University of Medical Sciences, Yasuj, Iran; ^6^ Department of Microbiology School of Medicine Yasuj University of Medical Sciences, Yasuj, Iran

## Abstract

**Materials and Methods:**

In a research experiment, 48 male Wistar rats were anesthetized and second-degree burns were induced on their backs. The rats' wounds were then uniformly inoculated with MRSA. Various treatments were applied to the burn wounds daily, including Myrtus ointment, silver nanoparticles, silver nanoparticles-Myrtus ointment, silver sulfadiazine-Myrtus ointment, silver sulfadiazine 1%, mupirocin ointment, and a positive control. The study measured the antimicrobial effects, wound area, percentage of wound healing, antioxidant capacities, malondialdehyde, and nitric oxide concentrations in the serum of the rats. Data analysis was performed using GraphPad software, with one-way ANOVA and Tukey's tests used to determine the statistical significance of the results.

**Results:**

Rats treated with Myrtus ointment, silver nanoparticles-Myrtus ointment, and mupirocin had reduced bacterial growth compared to the positive control group, nanoparticle ointment, and silver sulfadiazine (*P* < 0.05). The wound area of the Myrtus ointment group decreased significantly on the seventh and fourteenth days, as well as the level of MDA and nitric oxide, compared to the other groups. In Myrtus and silver sulfadiazine-Myrtus ointment increased the thickness of the epidermis and dermis compared to the other groups.

**Conclusion:**

Based on the anti-inflammatory, antimicrobial, and wound healing properties of Myrtus, with further studies, an ointment of this plant may be used as a main or complementary treatment for burn wound infections caused by MRSA.

## 1. Introduction

Burn injury is the fourth most common cause of injury in the world, accounting for 5 to 12% of hospital patients [1, 2]. Depending on the severity of the damage, the protective layers, which include the epidermis, dermis, and hypodermis, are damaged; therefore, the blood vessels in the dermis are destroyed and cause bleeding. Plasma, blood clots, and damaged protein structures in the layers of the skin are good places for microorganisms to enter. If the immune system fails to function properly, there is a risk factor for bacteremia and death. Approximately 75% of mortality from burns is due to complications of infection [3, 4]. *Pseudomonas aeruginosa* is the most important cause of burn infections. Then, *Staphylococcus aureus* is the second most important cause of infection in burns, and one of the main therapeutic challenges for these bacteria is that they quickly become resistant to antibiotics. Methicillin-resistant *Staphylococcus aureus* (MRSA) is one of the most important groups of *S. aureus* that is known as a health-threatening and challenging treatment. This bacterium has become resistant to methicillin by acquiring a gene called *mec*A, and its treatment is associated with several challenges. Quinolone resistance has also been reported in MRSA strains in recent years [5–7]. In fact, it seems that finding new therapies in combination with antibiotics is necessary to treat infectious agents in burns. Plant-based antimicrobial compounds have countless potentials in the treatment of infectious diseases, and sometimes their concomitant use with antibiotics has reduced the side effects of antibiotics [8]. The plant with the scientific name *Myrtus communis* (in Persian: Myrtus) from the Myrtaceae family is an evergreen plant that has bitter leaves with antibacterial, antioxidant, and antifungal medicinal properties [9, 10]. Some of its main active ingredients are quinoa, alpha-pinene, terpinol, and limonene, which are related to the antimicrobial properties of the plant. The oil of this plant is used in the treatment of infectious diseases due to its antioxidant, antimicrobial, and antibacterial properties [11, 12]. The results of Rezaie et al.'s study on the effects of this plant extract on wound healing in Wistar rats showed the complete healing of 22 mm wounds in rats receiving 5% leaf extract daily for about 24 days [13].

As mentioned, *S. aureus* is the second-most important cause of burn infections, and the antibiotic resistance of this bacterium, especially MRSA strains, has posed a serious challenge to the antibiotic treatment of burn infections. In addition, the antimicrobial effects of the Myrtus plant on *S. aureus* isolates, along with the low side effects of plants in antimicrobial treatments, suggest the use of ointments containing the extract of this plant as an option to heal infectious wounds caused by bacteria. Therefore, the aim of this study was to evaluate the effect of *Myrtus communis* leaf methanolic extract ointment and silver nanoparticles on the healing of burn wound infections induced by MRSA in rats.

## 2. Materials and Methods

### 2.1. Study Group

An experimental study was conducted in 2020 at Yasuj University of Medical Sciences with ethics code IR.YUMS.REC.1399.083 on 48 male Wistar rats weighing between 200 and 250 g on average. The study protocol was approved by the Ethics Committee for Animal Experiment at Yasuj University of Medical Sciences, School of Medicine in Yasuj, Iran.

### 2.2. Preparation of Methanolic Extract of *Myrtus communis*

*Myrtus communis* leaves were collected in the summer and dried in the shade. One hundred grams of ground leaves of the plant were placed in 1000 ml of 70% methanol at laboratory temperature for 48 hours. After 48 hours, Whatman no. 1 filter paper was used, and the container contents were passed through microbiology. The extract was poured into a sterile Pyrex container and dried at 40°C, then removed from the inner surface of the container and stored in sterile Falcon tubes at 4°C [14].

### 2.3. Synthesis of Silver Nanoparticles

The synthesis of nanoparticles was carried out using the green method, which involved using a methanolic extract of plant leaves. To do this, 60 mg of Myrtus leaf extract was dissolved in 1 ml of deionized water. First, 40 ml of 3 mM silver nitrate was placed on a hot plate machine at 1200 rpm and 80°C. Once the device reached the desired temperature, 1 ml of extract was added to it, and the solution was mixed for 30 minutes. The color change of the solution indicated the successful synthesis of silver nanoparticles. A centrifuge was used to separate the synthesized nanoparticles from the supernatant. The obtained silver nanoparticles were stored in microtubes at 4°C for further study [15].

### 2.4. Preparation of Ointment

Two types of ointments were prepared: a 5% Myrtus leaf ointment based on Eucerin and a 1% silver nanoparticle ointment. For the combination ointment of Myrtus and nanoparticles, 9.4 g of Myrtus extract was mixed with 0.1 g of nanoparticles and 95 g of Eucerin. In the combination ointment of silver sulfadiazine-Myrtus ointment, 50 g of 1% silver sulfadiazine ointment was mixed with 50 g of Myrtus ointment.

### 2.5. Animal Groups

In this experimental study, 48 male Wistar rats were randomly divided into 8 equal groups, including Group 1: negative control group: no burns without treatment; Group 2: positive control: with burns but without treatment; Group 3: daily recipient of 5% Myrtus ointment; Group 4: daily recipient of silver nanoparticle ointment; Group 5: daily recipient of silver nanoparticle ointment-Myrtus ointment; Group 6: daily recipient of 1% silver sulfadiazine ointment; Group 7: daily recipient of silver sulfadiazine-Myrtus ointment ointment; Group 8: daily recipient of mupirocin ointment. The groups receiving treatment were treated for 14 days.

### 2.6. Animal Tests

To induce burns, rats were first anesthetized intraperitoneally by injecting ketamine (50 mg/kg) and xylazine (50 mg/kg) according to their weight. The skin hairs on the back of the animal's lumbar region were completely shaved with a razor, and the skin was sterilized with 70% alcohol. Then, with the help of a heater with a temperature of 100 degrees and a duration of 10 seconds, a second-degree burn with an area of 3.14 cm^2^ was created on the back of the animal's neck. After burns, 2 cc of normal sterile saline was injected intraperitoneally to prevent hypovolemic shock. Wounds were cared for openly (for ointment groups), and the test animals were kept in separate cages. Twenty-four hours later, 0.1 cc of bacterial suspension (concentration: 1.5 × 10^7^) was injected into the burn site (in all mice) in several directions and subcutaneously and infection-induced. Direct culture was performed from the infected burn site with sterile swabs on days 1, 5, 7, 9, 12, and 14. To measure the level of burn on the first day of induction of burns and the seventh and fourteenth days, the radius of the wound was measured with a ruler, and the area of the wound was calculated. In the ointment treatment groups, the ointment was applied daily to the burn site with a sterile swab until the wound surface was covered. All animals were kept in a cycle of 12 hours of light and 12 hours of darkness, a temperature of 22 ± 2, and sufficient food and water. After the treatment period, the mice were anesthetized with ether, and blood was drawn from their hearts (1 to 3 cc). Their serum was separated and stored in a freezer at -80°C for biochemical tests. Half of each group was killed after deep anesthesia with a cervical vertebra on the seventh day for serum and tissue preparation and the other half on the 14th day.

The process of wound closure was evaluated by daily photography, and the quality of healing was evaluated by histological evaluation. The mice were anesthetized and dissected after blood sampling.

After washing with physiological serum, part of the tissue was kept in the microtube for homogenization and the other part was kept in a 10% formalin solution for 24 hours and then transferred to the new 5% formalin solution in the second 24 hours. Then 0.5 × 0.5 cm^2^ of each sample was cut and placed in a tissue processing machine. Tissue blocks were taken from the samples, and then using microtomes, 5-micron pieces were prepared from them and stained with hematoxylin-eosin dye. A Nikon light microscope and camera were used to examine them [16].

### 2.7. Biochemical Tests

#### 2.7.1. Measurement of Malondialdehyde

In this method, MDA (malondialdehyde) and TBA (thiobarbituric acid) affect each other, and the result is a pink complex. For this purpose, 100 *μ*l of the sample (serum and homogenized tissue) was diluted with 900 *μ*l of distilled water, and 100 *μ*l of TBA reagent was added to it. To prepare the TBA reagent, 100 ml of distilled water, 0.5 g of sodium hydroxide, 0.67 g of thiobarbituric acid, and 100 ml of acetic acid were mixed. The sample containing the reagent was heated at 90°C for one hour, and after cooling, the samples were centrifuged at 4000 g for 10 minutes. Then, the supernatant was separated, and the light absorption of the samples was read using a spectrophotometer at 535 nm. Based on comparison with the standard curve, the results were calculated and the amount of MDA was determined in terms of micromoles per milliliter [17].

#### 2.7.2. Nitric Oxide Assay

For protein degradation (with an acetonitrile solvent), 300 *μ*l of serum was mixed with 300 *μ*l of the solvent. Nitric oxide was measured by a grease reaction using the microplate method. To measure the concentration of nitrite and total nitrate, in an ELISA microplate, first 100 *μ*l of degreased serum and then 100 *μ*l of vanadium chloride solution (8 mg/ml) were added to convert the nitrates to nitrite. Then, 100 *μ*l of a 1 : 1 mixture of sulfonamides and NEDD was added and incubated for 30 minutes at 37°C. After the reaction and color formation, the light absorption of the color complex at 540 nm was read by the ELISA reader and the concentration of the samples was calculated using the standard curve [18].

#### 2.7.3. FRAP Assay (Ferric Reducing Antioxidant Power)

125 ml of acetate buffer solution, 12.5 ml of TPTZ, and 12.5 ml of ferric chloride were combined. Then, 1000 *μ*l of the above solution and 37 *μ*l of the sample were poured into 96-well plates, and its absorbance was read at 539 nm [19].

### 2.8. Statistical Analysis

Statistical analysis was performed using GraphPad software, and the results were reported as mean and standard deviation. Normality was determined before conducting one-way ANOVA and Tukey tests for statistical significance. The significance level of the tests was considered (*P* < 0.05).

## 3. Results

### 3.1. Comparison of Burn Area in the Studied Groups

**Figure 1:**
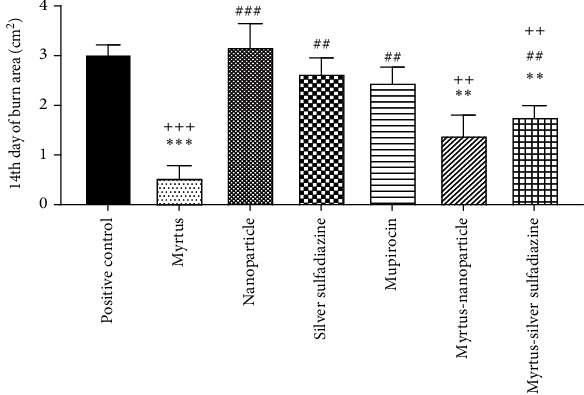
The effect of different treatment groups on the rate of wound healing on the 14th day in rats was as follows. ^∗^The positive control group showed a significant reduction in wound surface compared to all study groups (*p* < 0.01, *p* < 0.001). ^#^The Myrtus ointment group exhibited a significant decrease in wound surface compared to the other groups (*p* < 0.01, *p* < 0.001). ^+^The nanoparticle ointment group displayed a significant increase in wound surface compared to the Myrtus, Myrtus-nanoparticle, and silver sulfadiazine-Myrtus ointment groups (*p* < 0.01, *p* < 0.001). (++.^∗∗^, ##*p* < 0.01, +++.^∗∗∗^, ###*p* < 0.001).

According to the results of the statistical analysis, the wound area chart was drawn on days 0, 7, and 14 for all groups. On the seventh day, the wound area in the Myrtus ointment group was significantly reduced compared to all study groups (*P* < 0.05). In other words, treatment with the ointment reduced the wound area from 3.14 cm^2^ on day 0 to 1.58 cm^2^ on day 7 (Table 1). The wound area in the Myrtus ointment treatment group was 3.14 cm^2^ on the first day, which decreased to 0.53 cm^2^ on the fourteenth day (Table 1). In fact, on the 14th day, the wound level in the Myrtus ointment group decreased significantly compared to all groups and the silver sulfadiazine group compared to the positive control (*P* < 0.05) (Figure 1).

### 3.2. Comparison of Epidermal and Dermal Thickness

**Figure 2:**
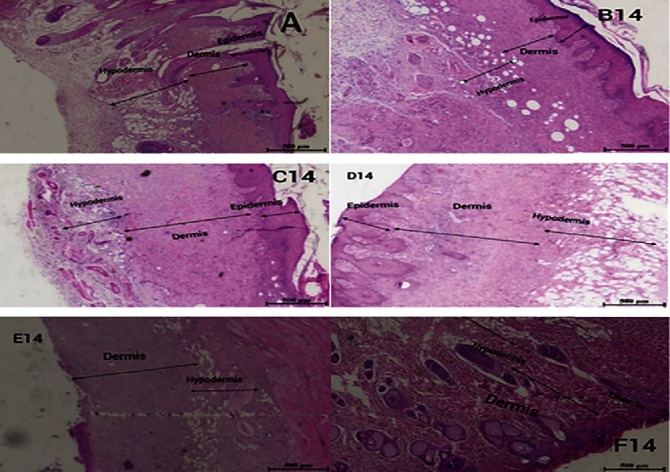
Comparison of skin wound healing process in different groups studied on 14th day in rats (H&E staining). (A) Positive control group; (B14) group treated with 5% Myrtus ointment; (C14) group treated with nanoparticle-Myrtus ointment; (D14) group treated with nanoparticle ointment; (E14) control group; (F14) treated with mupirocin ointment.

The thickness of the epidermis in all groups was zero on the seventh day, but on the fourteenth day, the thickness of the epidermis in Myrtus and silver sulfadiazine-Myrtus ointment was 312.5 *μ*m, and the silver sulfadiazine ointment group was 250 *μ*m (Figure 2). Figure 2 shows skin repair in the groups treated with Myrtus ointment, nanoparticle ointment, nanoparticle-Myrtus ointment, and mupirocin.

On the fourteenth day, the groups treated with Myrtus ointment, silver sulfadiazine-Myrtus ointment, silver sulfadiazine ointment, and mupirocin had a significant increase in dermis layer thickness in comparison with the positive control group (*P* < 0.05). On the other hand, the dermis layer thickness of the Myrtus ointment group was higher than other groups on the 14th day.

**Figure 3:**
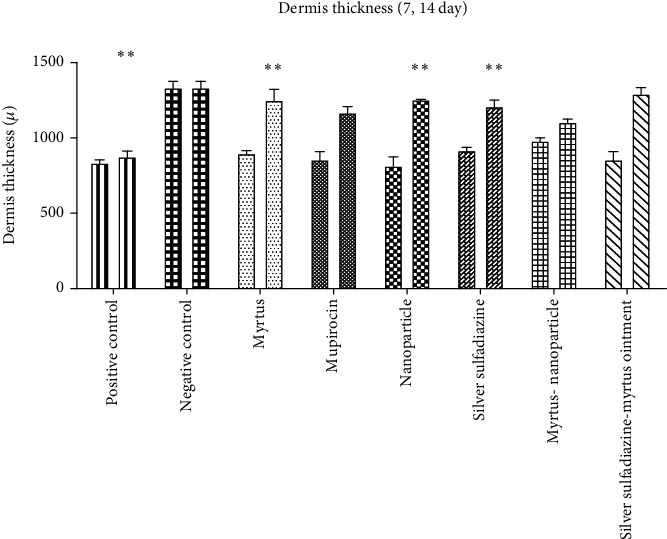
Dermis layer thickness was determined and compared with the untreated group in rats on the 7th and 14th days. The Myrtus, nanoparticle, silver sulfadiazine, and silver sulfadiazine-Myrtus ointment groups showed a significant increase in dermis thickness on the 14th day compared to the 7th day (^∗∗^*p* < 0.01). Additionally, the Myrtus, nanoparticle, silver sulfadiazine, mupirocin, and silver sulfadiazine-Myrtus ointment groups significantly increased dermis thickness on the 14th day compared to the positive control group on both the 7th and 14th days (^∗∗^*p* < 0.01).

Dermis thickness in the Myrtus ointment and silver sulfadiazine ointment groups was 875 *μ*m on the first day and 1375 *μ*m and 1250 *μ*m on the fourteenth day, respectively. The highest increases in dermis thickness were observed in the Myrtus ointment, silver sulfadiazine-Myrtus ointment, and mupirocin groups, respectively. On the seventh day, no significant difference in dermis thickness was observed between different groups (*P* > 0.05). On the fourteenth day, the Myrtus ointment group, silver sulfadiazine-Myrtus ointment, and mupirocin had a significant increase in dermis thickness compared to the positive control group (*P* < 0.05) (Figure 3). In histopathological results, dermal repair was observed in the Myrtus ointment-treated group compared to the control group (Figure 2). The process of repair and dermis formation in the ointment treatment groups suggests a better repair process.

### 3.3. Antimicrobial Results of the Studied Groups

**Figure 4:**
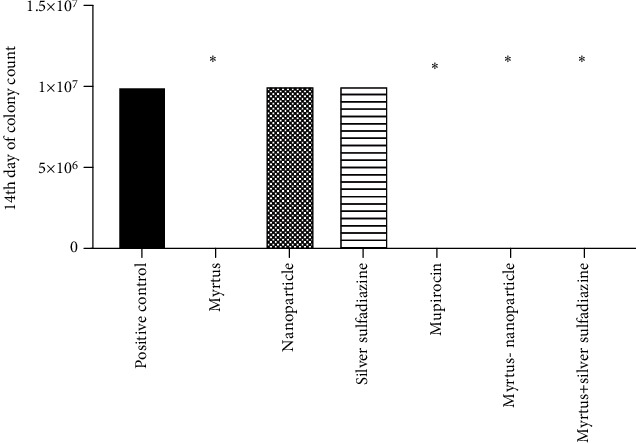
Antibacterial activity of Myrtus, Myrtus-nanoparticle, and silver sulfadiazine-Myrtus ointment against MRSA on the fourteenth day in rats. (^∗^*p* < 0.05).

The groups of mupirocin, Myrtus ointment, and silver sulfadiazine-Myrtus ointment significantly reduced bacterial colonies, respectively. On day 0, there was no significant difference between different groups (*P* > 0.05), but on the third day, the number of colonies in the groups of Myrtus ointment and mupirocin was significantly reduced compared to silver sulfadiazine ointment, silver sulfadiazine-Myrtus ointment, and the positive control group (*P* < 0.05). On the fifth, seventh, ninth, twelfth, and fourteenth days, the groups of Myrtus ointment, mupirocin, and silver sulfadiazine ointment had significantly reduced bacterial colonies compared to silver sulfadiazine ointment and the positive control (*P* < 0.0001) (Figure 4).

### 3.4. Biochemical Test

#### 3.4.1. Comparison of Serum Malondialdehyde (MDA)

**Figure 5:**
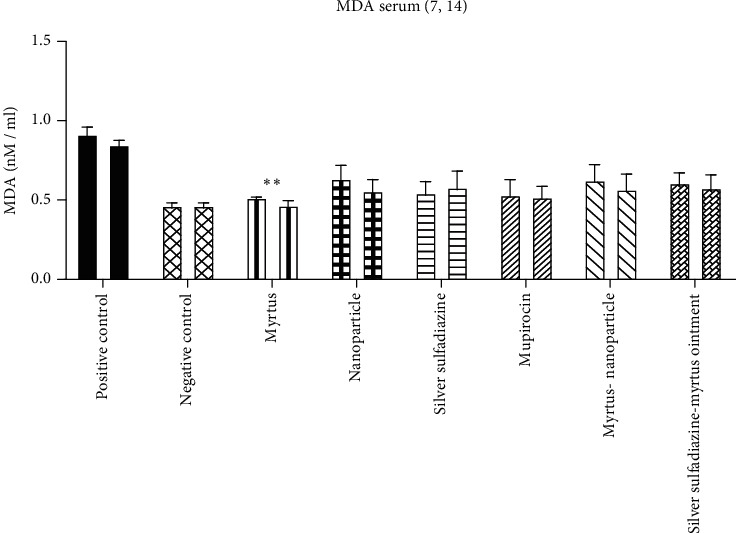
The effect of Myrtus on MDA levels in the serum of rats subjected to burn wounds. Myrtus ointment significantly reduced serum MDA compared to the positive control on the 14th day (^∗∗^*p* < 0.01).

Figure 5 shows that severe burns caused an obvious increase in the level of MDA in the serum of mice on the 7th and 14th days after the burn, and only in the Myrtus ointment group did it significantly decrease the MDA level compared to the positive control group on the 14th day (*P* = 0.01).

#### 3.4.2. Serum Nitrite Oxide Metabolites (Serum NO)

**Figure 6:**
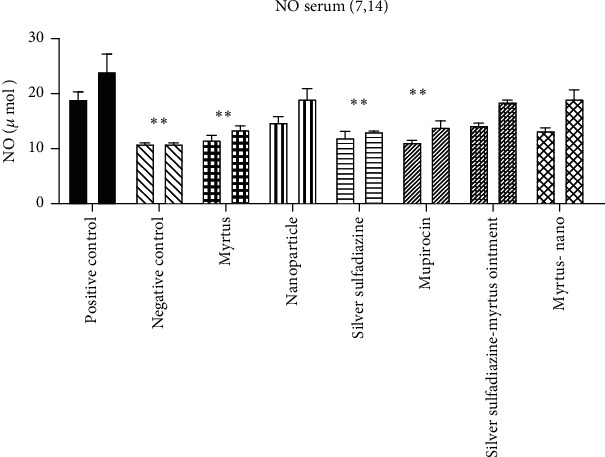
The effect of Myrtus on NO levels in the serum of rats subjected to burn wounds. High levels of NO reduced significantly by Myrtus on the 14th day. ^∗^Myrtus ointment, silver sulfadiazine, and mupirocin groups had a significant decrease in nitrite oxide compared to the positive control on the 14th day (^∗∗^*P* < 0.05).

Burn injury markedly increased the serum NO postburn. The amount of serum nitrite oxide metabolites in Myrtus, mupirocin, and silver sulfadiazine was significantly reduced compared to the positive control group on the 7th day. On the 14th day, the amount of serum NO in Myrtus and silver sulfadiazine was significantly reduced compared to the positive control (Figure 6).

#### 3.4.3. FRAP Assay

**Figure 7:**
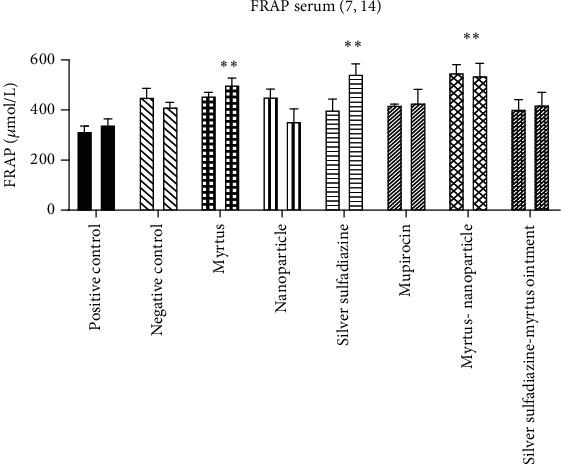
The effect of Myrtus, Myrtus-nanoparticles, and silver sulfadiazine on the total antioxidative potential of serum samples on days 7 and 14 in rats. Myrtus, Myrtus-nanoparticles, and silver sulfadiazine significantly increased compared to the positive control on day 14 (^∗∗^*P* < 0.05).

The burn induced an obvious reduction in the antioxidative potential in the rat's serum. In Myrtus, after treatment, the antioxidative potential in rat serum displayed a marked increase on the 7th day compared with the corresponding burn-positive control group. On the 14th day, Myrtus and silver sulfadiazine treatment groups significantly increased the antioxidative potential compared with those of the positive control group (Figure 7).

## 4. Discussion

Burns are one of the most devastating accidents that cause high mortality and high financial costs in society [20]. Due to the growing trend of antibiotic resistance, many efforts have been made to obtain more information about the effective use of plant compounds and their application in the treatment of various diseases. There has been a lot of research on the effect of medicinal plants on wound healing [21, 22]. In the present study, the results of wound measurements and histopathological evaluations indicate that on the seventh and fourteenth days after the burn, the wound area and time of complete healing were less in the 5% Myrtus ointment group than in the other groups. As we know, VEGF isoforms (vascular endothelial growth factors), which are regulated by HIF-1*α* protein, increase the rate of healing by creating angiogenesis at the burn site. Studies show that Myrtus significantly increases VEGF and HIF-1*α* proteins [23]. Therefore, the wound healing in the present study can be attributed to this issue. In a study conducted by Loghman et al., the restorative effects of the Myrtus plant extract on wound healing in Wistar rats were investigated as a topical ointment. In this study, it was found that complete wound healing in rats that received 10% ointment of Myrtus leaves daily took about 12 days [24]. In the present study, due to the use of 5% plant ointment, the complete recovery time in the Myrtus ointment group was 14 days.

In the present study, the Myrtus ointment inhibited the growth of MRSA at the wound site. The mechanism of the antimicrobial effect of the plant may be related to its polyphenolic compounds, two important components of which are myrtucommulone A and B. These two substances have antimicrobial effects against gram-positive bacteria [25]. In another study, Reddy et al. examined the antibacterial activity of extracts of eight different medicinal plants against MRSA by disk diffusion test and broth microdilution test (determination of minimum inhibitory concentration). The MIC of *Myrtus communis* against MRSA was 50 mg/ml. In our study, it was 4 mg/ml, which is probably due to differences in plant species or differences in the genetic nature of bacteria [26].

The results of the present study indicated that the burn induced an obvious increase in serum malondialdehyde and nitric oxide and decreased serum antioxidative potential. On the fourteenth day, in the group treated with Myrtus leaf extract ointment, a decrease in MDA and NO and an increase in antioxidative potential were observed. In a study conducted by Falleh et al., which examined the effect of the plant Myrtus on the burn, it was found that the level of MDA in the burn group increased significantly compared to the control group. Both oral and topical treatments of *Myrtus communis* significantly reduced the amount of MDA compared to the burn group [27]. Nitric oxide is an active radical whose high amounts cause tissue damage, so plants that can counteract the formation of nitric oxide are considered a factor in disease control. The body's antioxidant defense system is a mechanism to counteract the effects of free radicals. Without such a mechanism in the body, these compounds, with their high potential, destroy large biological molecules such as fats, DNA, and proteins [28, 29]. Studies showed that *Myrtus communis*, rich in tannins, flavonoids, and vitamin C, which increase antioxidant defense, eliminates the final product of lipid peroxidation (MDA) and NO free radicals [30, 31]. Therefore, one of the reasons for the antioxidant properties of *Myrtus communis* in the present study may be associated with these compounds [10]. Najafi et al. evaluated the effect of *Myrtus communis* L. extract on several basic mechanisms effective in wound healing and stated that the extract of this plant significantly reduces the expression of iNOS and thus reduces NO. As a result, this plant can reduce oxidative stress and has very strong antioxidant properties [30]. The results of our study are consistent with the above studies, and the extract of *Myrtus communis* reduced nitric oxide and increased the antioxidative potential.

## 5. Conclusion

**Figure 8:**
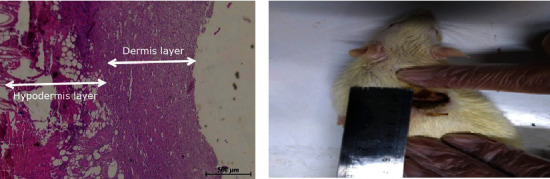
The healing process of skin wounds in Myrtus ointment groups was studied on the 7th day in rats (H&E staining).

Based on the anti-inflammatory, antimicrobial, and wound healing properties of the Myrtus plant, with further studies, the ointment of this plant can be used as the main or complementary treatment for burn wound infections caused by MRSA (Figure 8).

## Figures and Tables

**Table 1:** Mean ± standard deviation of burn area on the 7th and 14th days in different studied groups.

Groups	Burn area (cm^2^)		
	Day zero	Seventh days	Fourteenth days
Myrtus ointment	3.14 ± 0	1.58 ± 0.18	0.53 ± 0.24
Silver nanoparticle ointment	3.14 ± 0	3.72 ± 0.53	3.17 ± 0.47
Silver nanoparticle ointment and Myrtus ointment	3.14 ± 0	2.69 ± 0.30	1.39 ± 0.41
Mupirocin ointment	3.14 ± 0	2.82 ± 0	2.28 ± 0.53
Silver sulfadiazine ointment	3.14 ± 0	2.82 ± 0.22	2.62 ± 0.32
Myrtus ointment and silver sulfadiazine ointment	3.14 ± 0	2.82 ± 0	1.76 ± 0.23
Positive control	3.14 ± 0	3.14 ± 0	3.03 ± 0.18

## Data Availability

You can get the information from the corresponding author.
